# Supplementary figures and images for: Keratin 15 promotes a progenitor cell state in basal keratinocytes of skin epidermis
Source: J Cell Biol. 2026 Jan 9;225(3):e202503046. doi: 10.1083/jcb.202503046 (PMC12784932; doi:10.1083/jcb.202503046)

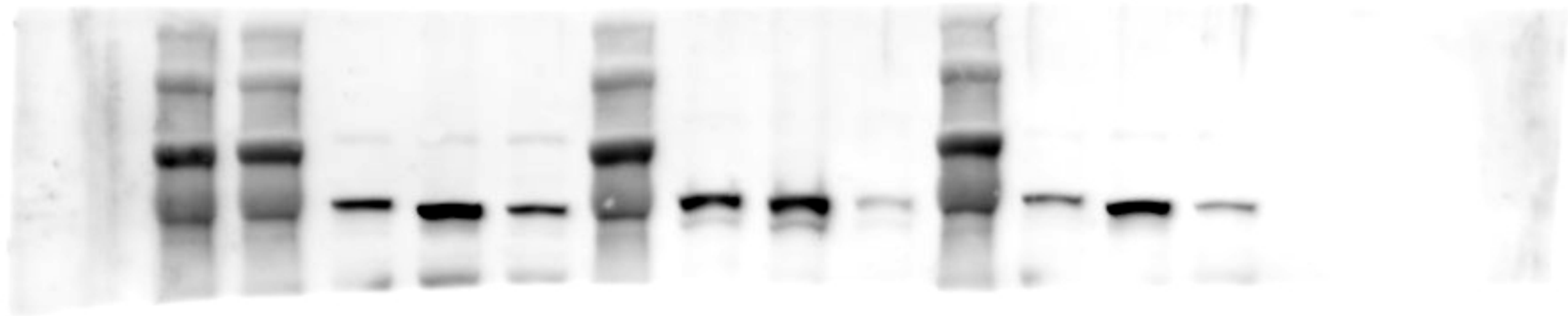

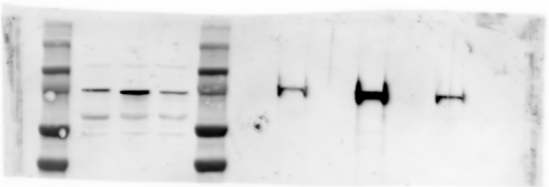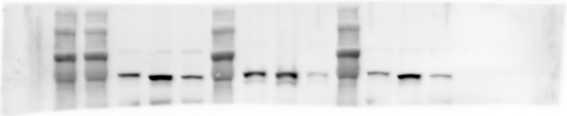

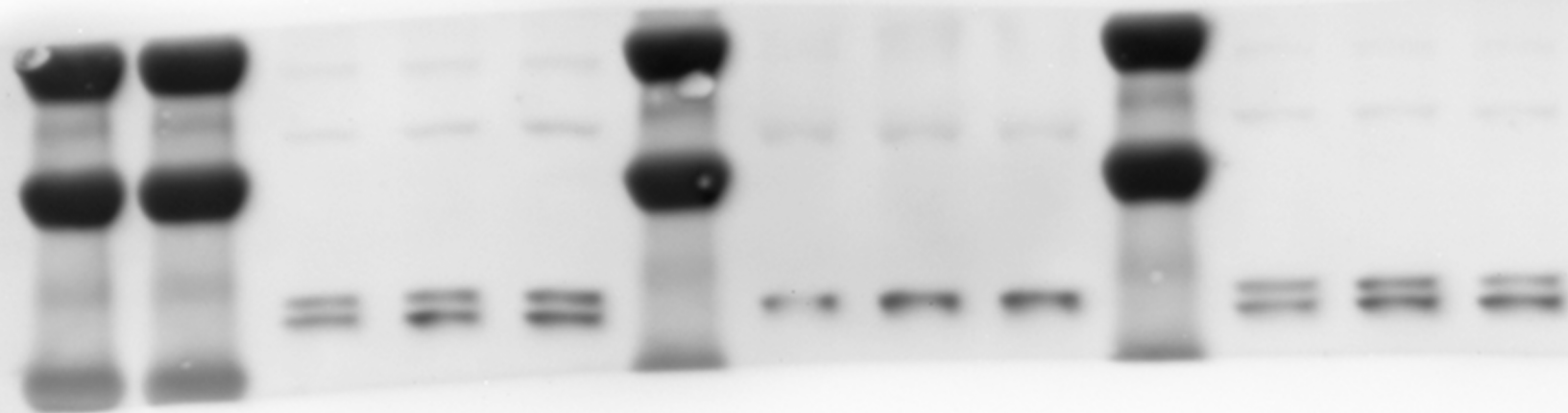

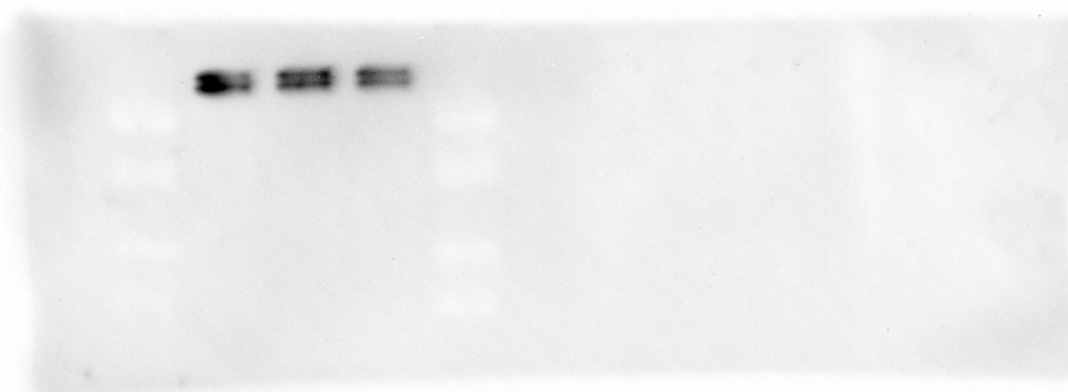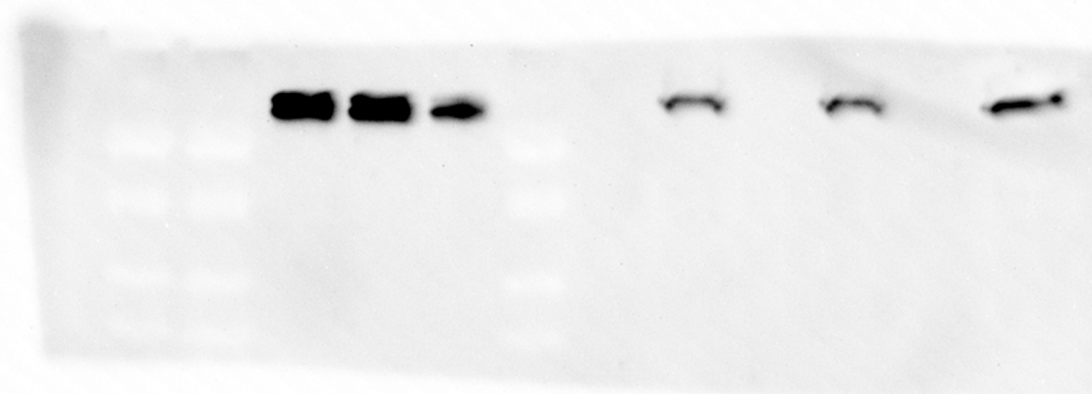

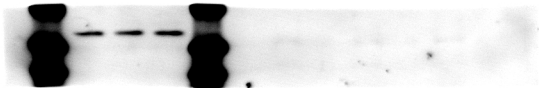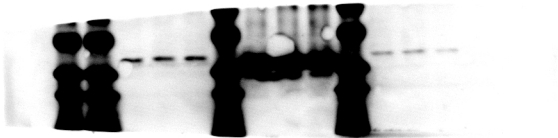

Supplement: SourceData F6 — is the source file for Fig. 6. [file jcb_202503046_sourcedataf6.pdf]
